# Are Both Ultrasonography and Mammography Necessary for Cancer Investigation of Breast Lumps in Resource-Limited Countries?

**DOI:** 10.1155/2013/257942

**Published:** 2013-08-28

**Authors:** Rungnapa Chairat, Adisorn Puttisri, Asani Pamarapa, Sahatham Samintharapanya, Chamaiporn Tawichasri, Jayanton Patumanond

**Affiliations:** ^1^Program in Clinical Epidemiology, Faculty of Medicine, Chiang Mai University, Chiang Mai 50200, Thailand; ^2^Department of Nursing, Uttaradit Hospital, Uttaradit 53000, Thailand; ^3^Department of General Surgery, Nakon Swan General Hospital, Nakon Swan 60000, Thailand; ^4^Department of General Surgery, Uttaradit General Hospital, Uttaradit 53000, Thailand; ^5^Department of General Surgery, Lampang General Hospital, Lampang 52000, Thailand; ^6^Clinical Epidemiology Society, Chaing Mai 50200, Thailand; ^7^Clinical Epidemiology Unit, Faculty of Medicine, Chiang Mai University, Chiang Mai 50200, Thailand

## Abstract

*Objective*. To reevaluate the diagnostic value of breast imaging in the diagnosis of breast cancer in areas where health resources are limited. *Methods*. Patients were women presenting with breast lumps in two university-affiliated tertiary hospitals, Thailand, during 2006 and 2010. Clinical data were abstracted from the breast cancer registration database and patient records. The diagnostic predictive ability of ultrasonography and mammography was obtained from logistic regression analysis and presented with areas under the receiver operating characteristics (AuROCs) curves. *Results*. Among 3129 breast lumps (3069 women), 854 were diagnosed with breast cancer by certified pathologists. Age and size of lumps alone already predicted cancer correctly in 77.45% (AuROC = 77.45). Additional ultrasonography increased the prediction to 96.22% (*P* < 0.001). Additional mammography also increased the prediction to 95.99% (*P* < 0.001). Performing both imaging modalities did not increase the prediction clinically (0.01%–0.24%). More accurate prediction (2.07%–2.21%) may be added by fine needle aspiration cytology (FNAC). *Conclusions*. Breast imaging is still valuable in settings where health resources are limited. Single breast imaging (only either ultrasonography or mammography) is adequate for cancer diagnosis. It is therefore unnecessary to perform both imaging modalities. Accuracy of the diagnosis may be improved by FNAC, if available.

## 1. Introduction 

Breast cancer is increasingly common in women worldwide. It is one of the leading causes of death among female malignancies [1]. The incidence varies from region to region, more in developed countries (>80 per 100,000 populations) than in developing countries (<40 per 100,000 populations). In southeast Asian women, it was 31.0 per 100,000 populations [2]. Cancer of the breast in Thailand is the highest among all female cancers and is continuously rising. The incidence increased from 34.4 to 40.6 per 100,000 populations during 2006 to 2009. The mortality also increased from 6.3 to 7.3 per 100,000 populations in the corresponding years [3].

The standard diagnosis of breast cancer recommended triple diagnostic investigations [4], comprising (1) clinical breast examination; (2) breast imaging, ultrasonography, or mammography, (3) cytopathologic with fine needle aspiration cytology (FNAC) or histopathologic findings with a core needle biopsy with cutting needle biopsy. The same recommendation was also used by The National Institute of Cancer of Thailand, for women presenting with breast lumps [5]. Ultrasonography is recommended in women under 35 years old, and both ultrasonography and mammography are recommended in those 35 years old or above. Although breast images have been proved effective in diagnosing cancer in women presenting with breast lumps [6], their uses are, however, limited, either by lack of trained medical personnel or their unavailability. Referral of patients to better equipped hospitals only for imaging investigations may somehow cause the delay in diagnosis and treatments [7].

In routine practice, physicians in countries with limited resources approach women with breast lumps with history taking clinical breast examination and breast pathology by FNAC or biopsy and skipped breast imaging (mammography or ultrasonography) to shorten the time of diagnosis and treatment. However, clinical breast examination alone lacks reliability for cancer diagnosis [8]. Although researches indicated that diagnosis of cancer may be adequately made by FNAC [7], the results may still contain false-negatives and false-positives [9]. The procedure (FNAC) also cannot differentiate in situ from invasive breast cancer. There are still not enough evidences to conclude which investigations should be used when considering effectiveness together with availability, leading to varying recommendations and guidelines [10].

The present study evaluated the ability of breast imaging (mammography and ultrasonography) in diagnosing cancer in women presenting with breast lumps, in the presence of patient profiles, with or without FNAC. The study also considered their combinations, sequences, and availability of each investigative modality, in order to reflect situations where health resources are limited.

## 2. Subjects and Methods

### 2.1. Subjects and Settings

Patients were women who presented with breast lumps in two university-affiliated tertiary care hospitals, Lampang Hospital and Uttaradit Hospital, located in the northern part of Thailand. Patient medical records between 2006 and 2010 were retrieved under the ICD-10 specification. Case tracing was made for at least 12 months after the first presentation to the hospitals to verify any changes in final diagnosis. Women with a history of breast biopsy, or breast silicone implantation were not included. In this nested case-control analogue design, cases were women verified as cancer by breast pathology (biopsy or operative specimen). Controls were those with noncancerous lumps proved by the same criteria or clinical breast examination and/or breast imaging and/or FNAC.

### 2.2. Data and Source

The keyed information, age (years), body mass index (BMI, kg/m^2^), duration of lumps (month), size of lumps (cm), side of lumps, quadrant, clinical characteristics, and final histopathology, was retrieved from the medical registration and patient records.

### 2.3. Diagnostic Investigations

For breast imaging results, mammography and ultrasonography were interpreted by the hospital radiologists under routine practices. The classification of breast imaging followed the BI-RADS (Breast Imaging Reporting and Data System) [11] (Table 1).

Cytopathologic diagnosis was verified by certified university hospital pathologists. FNAC classification followed the Cytologic Category Code System [12, 13] (Table 1).

### 2.4. Data Analysis

General characteristics and clinical characteristics were presented with descriptive statistics and compared between patients with and without cancer by exact probability tests, *t*-tests, or Wilcoxon's rank sum tests, as appropriate.

Logistic regression techniques were used to obtain diagnostic values of age, size, imaging techniques, and FNAC and presented with diagnostic odds ratios (dORs), 95% confidence interval (CI), and *P* value.

The diagnostic abilities of the 3 main diagnostic investigations (ultrasonography, mammography, and FNAC) in combination with clinical baseline (age and size of lumps) were compared with the clinical baseline alone, by area under the receiver operating characteristics (AuROCs) curves.

The differences in predictive abilities of diagnostic modality were compared by the likelihood ratio tests (LR tests) under the logistic models. Application utilities of the investigation sequences were calculated by predictive ability (%) times availability (%) to guide clinical approaches in real situations.

## 3. Results

From 3129 breast lumps (3069 patients), there were 854 lumps with definite (postoperative histopathology) diagnosis of breast cancer and 2275 lumps without. Review of the index investigation, ultrasonography, mammography, and FNAC showed significant different results between those with and without cancer (*P* < 0.001) (Table 1).

 Patients were between 15 and 93 years old (mean = 45.8 ± 12.8). Those with cancer were older (53.2 ± 11.6 versus 43.1 ± 12.0 years, *P* < 0.001) and had higher body mass index (23.7 ± 6.2 versus 22.7 ± 2.2 kg/m^2^, *P* = 0.003). The duration of noticing breast lumps was shorter (4.0 ± 9.9 versus 4.4 ± 14.0 months, *P* < 0.001). Lump sizes ranged from 0.5 to 15 cm, larger in those with cancer (3.1 ± 1.9 versus 1.6 ± 1.1 cm, *P* < 0.001). More than half of the lumps were palpable in the upper outer quadrants. Right and left sided lumps were similar in both groups. Fixed lumps, retracted skin, and retracted nipple were more frequent in those with cancer (*P* < 0.001) (Table 2).

The independent predictive ability of the three investigative modalities in the presence of baseline information (age and size) under the multivariable logistic regression showed high diagnostic odds ratios of 3.85 for ultrasonography, 2.08 for mammography, and 3.52 for FNAC (Table 3).

**Figure 1:**
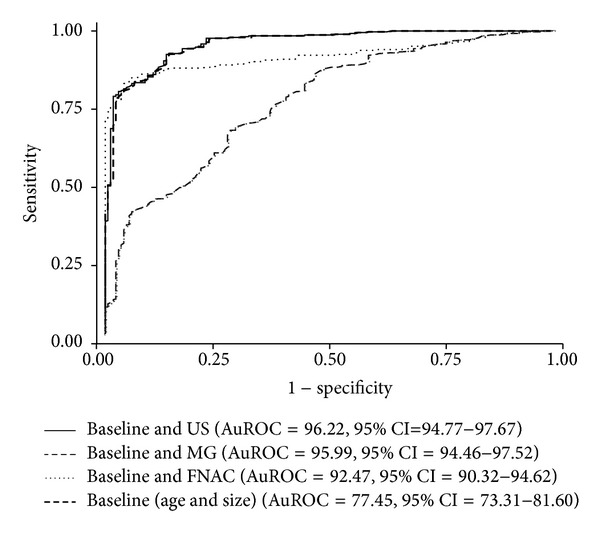
Comparative area under receiver operating characteristic (AuROC) curves of additional diagnostic investigation; ultrasonography (US), mammography (MG), and fine needle aspiration cytology (FNAC) on top of baseline (age with size).

Baseline information, age and lump sizes, alone predicted cancer correctly in 77.45% (AuROC = 77.45, 95% CI = 73.31–81.60). Single investigation on top of baseline showed the highest to the lowest prediction for ultrasonography (AuROC = 96.22%, 95% CI = 94.77–97.67), mammography (AuROC = 95.99%, 95% CI = 94.46–97.52), and FNAC (AuROC = 92.47%, 95% CI = 90.32–94.62) (Figure 1).

**Figure 2:**
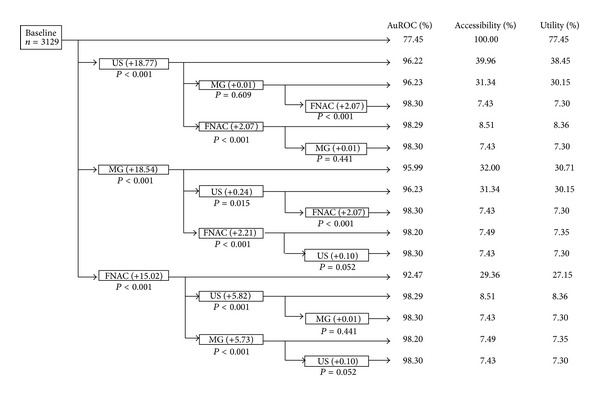
Comparative area under receiver operating characteristic (AuROC) curves, additional diagnostic value, accessibility, and utility of diagnostic investigation; ultrasonography (US), mammography (MG), and fine needle aspiration cytology (FNAC) on top of baseline (age with size).

Modality combination with the highest prediction was breast imaging (either ultrasonography or mammography) with FNAC. FNAC after ultrasonography added 2.07% (*P* < 0.001). FNAC after mammography also increased the prediction by 2.21% (*P* < 0.001). Ultrasonography after mammography added very little prediction (0.24%, *P* = 0.015). Ultrasonography followed by mammography also added very little prediction (0.01%, *P* = 0.609) (Figure 2).

The investigative modality sequence that yielded high additional prediction leading to high final prediction was breast imaging (either ultrasonography or mammography) followed by FNAC.

## 4. Discussion

Breast imaging (either ultrasonography or mammography) increased cancer prediction beyond baseline information with AuROCs of 96.22% and 95.99%. Their utilities were among the highest (38.45% and 30.71%). Previous studies also reported high reliability of the two modalities, but there were no reports on availability or utility in routine practice, nor reports on appropriate combination or sequences of investigations [6, 14–18]. It is therefore difficult to compare diagnostic accuracies of investigative modalities across studies [7].

In the area with restricted health resources, clinicians may diagnose breast cancer using clinical breast examination and FNAC, without any of breast imaging modalities. The present study agreed that clinical breast examination alone was not adequate for cancer diagnosis [8, 19]. Although FNAC on top of baseline information might be efficient (AuROC = 92.47%, OR = 3.52, *P* < 0.001) [7, 20–23], but false-negative results from FNAC may lead to delayed and/or under treatment, while false-positive results may lead to over treatment [24]. Additional ultrasonography or mammography after FNAC increases cancer prediction by 5.82% and 5.73%, clinically and statistically significant, but its application utility decreased profoundly to 8.36% and 7.30%.

Comparing between the two imaging modalities, ultrasonography had higher cancer prediction and utility than mammography. Mammography may have some limitations in diagnosing dense breast [25, 26], while ultrasonography may have more advantages in diagnosing breast cyst. The accuracy of ultrasonography in diagnosing cyst was as high as 92%, but only 65% in diagnosing cancer [27]. Ultrasonography may also detect cancer from stage 1 to stage 4. Microcalcification was detected in 74% of patients with ductal carcinoma in situ of the breast (54 in 73 patients) [28]. Ultrasonography also detected abnormality when mammography reported Bi-RADS = 0 [29]. Subgroup analysis also showed that patients with Bi-RADS below 4 might gain more benefit from additional ultrasound investigation, while for those with Bi-RADS 4 and above who already had high possibility for cancer, ultrasound might not gain any additional information.

The findings indicated that breast imaging was valuable in diagnosing cancer in patients presenting with breast lumps with high utility. In health restricted resource countries, performing both imaging modalities (ultrasonography and mammography) may be unnecessary [30]. However, a better prediction may be gained by a combination of different modalities ultrasonography followed by FNAC, or mammography followed by FNAC, depending on availability and/or preference.

Breast imaging investigation should therefore be encouraged and made available countrywide in order to reduce cancer mortality in women presenting with breast lumps suspicious of breast cancer [10].

## 5. Conclusions

Breast imaging, either ultrasonography or mammography, had high additional diagnostic value of cancer prediction over baseline in patients presenting with breast lumps. However, both mammography and ultrasonography may be unnecessary. Which modality should be performed first was due to its availability and/or center preferences. However, additional FNAC, if available, may increase cancer prediction, both clinically and statistically.

## Figures and Tables

**Table 1:** Classification of breast imaging (ultrasonography and mammography) and fine needle aspiration cytology (FNAC) investigations (3069 subjects, 3129 lumps).

Classifications	Interpretation	Malignant		Benign		*P* value
		*n*	%	*n*	%	
Ultrasonography						
bi-rads 0	Need additional imaging evaluation	0	0	4	0.3	<0.001
bi-rads 1	Negative	0	0	116	8.2	
bi-rads 2	Benign	14	2.2	964	68.0	
bi-rads 3	Probably benign	20	3.2	240	16.9	
bi-rads 4	Suspicious for abnormality	164	25.9	86	6.1	
bi-rads 5	Highly suggestive of malignancy	435	68.7	7	0.5	
Mammography						
bi-rads 0	Need additional imaging evaluation	0	0	2	0.2	<0.001
bi-rads 1	Negative	0	0	88	7.4	
bi-rads 2	Benign	14	2.2	815	68.4	
bi-rads 3	Probably benign	20	3.2	198	16.6	
bi-rads 4	Suspicious for abnormality	162	25.8	81	6.8	
bi-rads 5	Highly suggestive of malignancy	432	68.8	7	0.6	
FNAC						
class 1	Inadequate, unsatisfactory	47	9.5	86	18.4	<0.001
class 2	Benign cell present	17	3.5	356	76.1	
class 3	Probably benign	76	15.5	24	5.1	
class 4	Suspicious for malignancy	67	13.6	2	0.4	
class 5	Malignant cell present	285	57.9	0	0	

**Table 2:** General characteristics of patients (3069 subjects, 3129 lumps).

Characteristics	Malignant		Benign		*P* value
	mean	(SD)	mean	(SD)	
Age (years)	53.2	(11.6)	43.1	(12.0)	<0.001
BMI (kg/m^2^)	23.7	(6.2)	22.7	(2.2)	0.003
Duration (month)	4.0	(9.9)	4.4	(14.0)	<0.001
Size (cm)	3.1	(1.9)	1.6	(1.1)	<0.001
Side (*n*, %)					
Right	419	49.1	1,102	48.4	0.779
Left	435	50.9	1,173	51.6	
Quadrant (*n*, %)					
Upper outer	467	54.7	1,157	50.9	0.121
Upper inner	188	22.0	556	24.4	
Subareolar	55	6.5	145	6.4	
Lower outer	95	11.1	239	10.5	
Lower inner	49	5.7	178	7.8	
Clinical manifestations (*n*, %)					
Fixed lump	346	47.1	40	2.5	<0.001
Retracted skin	30	4.5	9	0.7	<0.001
Nipple discharge	19	2.8	41	2.7	1.000
Rugged edge	74	10.9	141	10.3	0.702
Wound/ulcer	13	2.0	26	2.1	1.000

**Table 3:** Independent effects of clinical parameters used in logistic modelling for breast cancer diagnosis, measured as multivariable diagnostic odds ratio (dOR).

Parameters	dOR	95% Confidence interval	*P* value
Age (per year)	1.02	0.98 to 1.06	0.313
Size (per cm)	1.77	1.23 to 2.54	0.002
Ultrasonography (per bi-rads)	3.85	0.80 to 18.43	0.092
Mammography (per bi-rads)	2.08	0.44 to 9.90	0.359
FNAC (per class)	3.52	2.45 to 5.08	<0.001
